# Translating virome analyses to support biosecurity, on-farm management, and crop breeding

**DOI:** 10.3389/fpls.2023.1056603

**Published:** 2023-03-14

**Authors:** Ricardo I. Alcalá Briseño, Ozgur Batuman, Jeremy Brawner, Wilmer J. Cuellar, Erik Delaquis, Berea A. Etherton, Ronald D. French-Monar, Jan F. Kreuze, Israel Navarrete, Kwame Ogero, Aaron I. Plex Sulá, Salih Yilmaz, Karen A. Garrett

**Affiliations:** ^1^ Plant Pathology Department, University of Florida, Gainesville, FL, United States; ^2^ Global Food Systems Institute, University of Florida, Gainesville, FL, United States; ^3^ Emerging Pathogens Institute, University of Florida, Gainesville, FL, United States; ^4^ Plant Pathology Department, Oregon State University, Corvallis, OR, United States; ^5^ Southwest Florida Research and Education Center (SWFREC), Immokalee, FL, United States; ^6^ International Center for Tropical Agriculture (CIAT), Cali, Colombia; ^7^ International Center for Tropical Agriculture (CIAT), Vientiane, Laos; ^8^ USDA-APHIS-PPQ-FO Plant Germplasm Quarantine Program (PGQP), Laurel, MD, United States; ^9^ Crop and System Sciences Division, International Potato Center (CIP), Lima, Peru; ^10^ Crop and System Sciences Division, International Potato Center (CIP), Quito, Ecuador; ^11^ Crop and System Sciences Division, International Potato Center (CIP), Mwanza, Tanzania

**Keywords:** crop breeding, microbiomes, pest management, phytosanitary standards, seed systems, surveillance, viromes

## Abstract

Virome analysis *via* high-throughput sequencing (HTS) allows rapid and massive virus identification and diagnoses, expanding our focus from individual samples to the ecological distribution of viruses in agroecological landscapes. Decreases in sequencing costs combined with technological advances, such as automation and robotics, allow for efficient processing and analysis of numerous samples in plant disease clinics, tissue culture laboratories, and breeding programs. There are many opportunities for translating virome analysis to support plant health. For example, virome analysis can be employed in the development of biosecurity strategies and policies, including the implementation of virome risk assessments to support regulation and reduce the movement of infected plant material. A challenge is to identify which new viruses discovered through HTS require regulation and which can be allowed to move in germplasm and trade. On-farm management strategies can incorporate information from high-throughput surveillance, monitoring for new and known viruses across scales, to rapidly identify important agricultural viruses and understand their abundance and spread. Virome indexing programs can be used to generate clean germplasm and seed, crucial for the maintenance of seed system production and health, particularly in vegetatively propagated crops such as roots, tubers, and bananas. Virome analysis in breeding programs can provide insight into virus expression levels by generating relative abundance data, aiding in breeding cultivars resistant, or at least tolerant, to viruses. The integration of network analysis and machine learning techniques can facilitate designing and implementing management strategies, using novel forms of information to provide a scalable, replicable, and practical approach to developing management strategies for viromes. In the long run, these management strategies will be designed by generating sequence databases and building on the foundation of pre-existing knowledge about virus taxonomy, distribution, and host range. In conclusion, virome analysis will support the early adoption and implementation of integrated control strategies, impacting global markets, reducing the risk of introducing novel viruses, and limiting virus spread. The effective translation of virome analysis depends on capacity building to make benefits available globally.

## Introduction to the plant virome

Advances in high-throughput sequencing (HTS) technologies have expanded our understanding of virus communities and their impacts on ecosystems, including the ongoing COVID-19 pandemic (Fauci et al., 2020; CDC, 2022). Emerging plant viruses are a threat to crops globally. Advances in plant virus ecology are needed to fully understand the effects of global change such as agricultural intensification and climate change, and how these interact with virus traits such as mode of transmission (Adams et al., 2014; Salem et al., 2016; Jeger, 2020; Siriwan et al., 2020; Uke et al., 2021). Interactions between virus species are another major epidemic driver (Syller, 2011; Tollenaere et al., 2016). An important new phase of the study of virus communities is putting this information to work to support better agriculture. Translating analyses of virus communities to support effective management strategies depends on integrating HTS data and agricultural system knowledge.

The virome concept emerged in parallel with the general microbiome concept (Wylie et al., 2012; Berg et al., 2020). Virome analyses can include the study of viruses in a single host, or at multiple scales, such as host plants or vector species, the set of hosts within a location, or environmental samples such as from water or soil (Kreuze et al., 2009; Al Rwahnih et al., 2011; Rosario et al., 2015; Leke et al., 2016; Hadad et al., 2019; Wylie et al., 2019; Alcalá-Briseño et al., 2020; Bacnik et al., 2020; Britt et al., 2020; Kwok et al., 2020; Fontenele et al., 2021). Implementing HTS allowed virome analysis to thrive through viral metagenomics, combined with new sampling options (i.e., individual, bulked, and mixed samples) and expanded virus sequence databases. Our understanding of viromes is rapidly advancing, including viral diversity, genetic composition, genomic organization, and phylogenetic relationships among virus species in ecosystems (Kreuze et al., 2009; Al Rwahnih et al., 2011; Leke et al., 2016; Wylie et al., 2019). The virome perspective expanded our understanding of how viruses and plants interact, building on knowledge about the most prevalent and well-described viruses, which are often the most damaging to current crop systems. A frontier for epidemiology is understanding the interactions and antagonistic effects of virus species and their vectors, especially for viruses that produce subtle or no visual symptoms when infecting alone (Jeger et al., 2023). Most studies focus on economically important crops, but secondary hosts may function as reservoirs for crop viruses, and virus spillover may occur between natural and crop ecosystems (Alexander et al., 2013; Bernardo et al., 2017; Ingwell et al., 2017; Alcalá-Briseño et al., 2020). Studying the dynamics of viruses between crops and weeds is necessary for implementing effective management strategies. Understanding these interactions at a large scale in complex agroecological systems may inform effective management strategies.

Climate change is shifting the distribution of plant-host and insect-vector populations (Jones, 2016; Bebber et al., 2019; Chaloner et al., 2021; Wang et al., 2022). The connectivity of the agricultural landscape also influences the introduction and dispersal of pathogens, often increasing the risk of global pathogen emergence (Fahrig et al., 2011; Garrett et al., 2020; Xing et al., 2020; Jones, 2021). Scientists and practitioners must consider pathogen risks and societal impacts to effectively address and mitigate these challenges. There are a wide range of new tools and frameworks for improving virome data analysis, virus discovery and diagnostics, and applications such as phytosanitary practices and regulation (Massart et al., 2014; Massart et al., 2017; Massart et al., 2019; Maclot et al., 2020; Kumar et al., 2021; Moubset et al., 2022). Our goal in this paper is to synthesize current knowledge of virus discovery and state-of-the-art technologies, and the potential for translation of big data to benefit stakeholders, from national agencies to individual farmers. Approaches such as machine learning and network analysis can inform translation of virome analysis into management strategies to mitigate the effects of plant pathogens in a changing world.

### Translating virome analysis: From ecology and molecular analysis to surveillance and management

**Figure 1:**
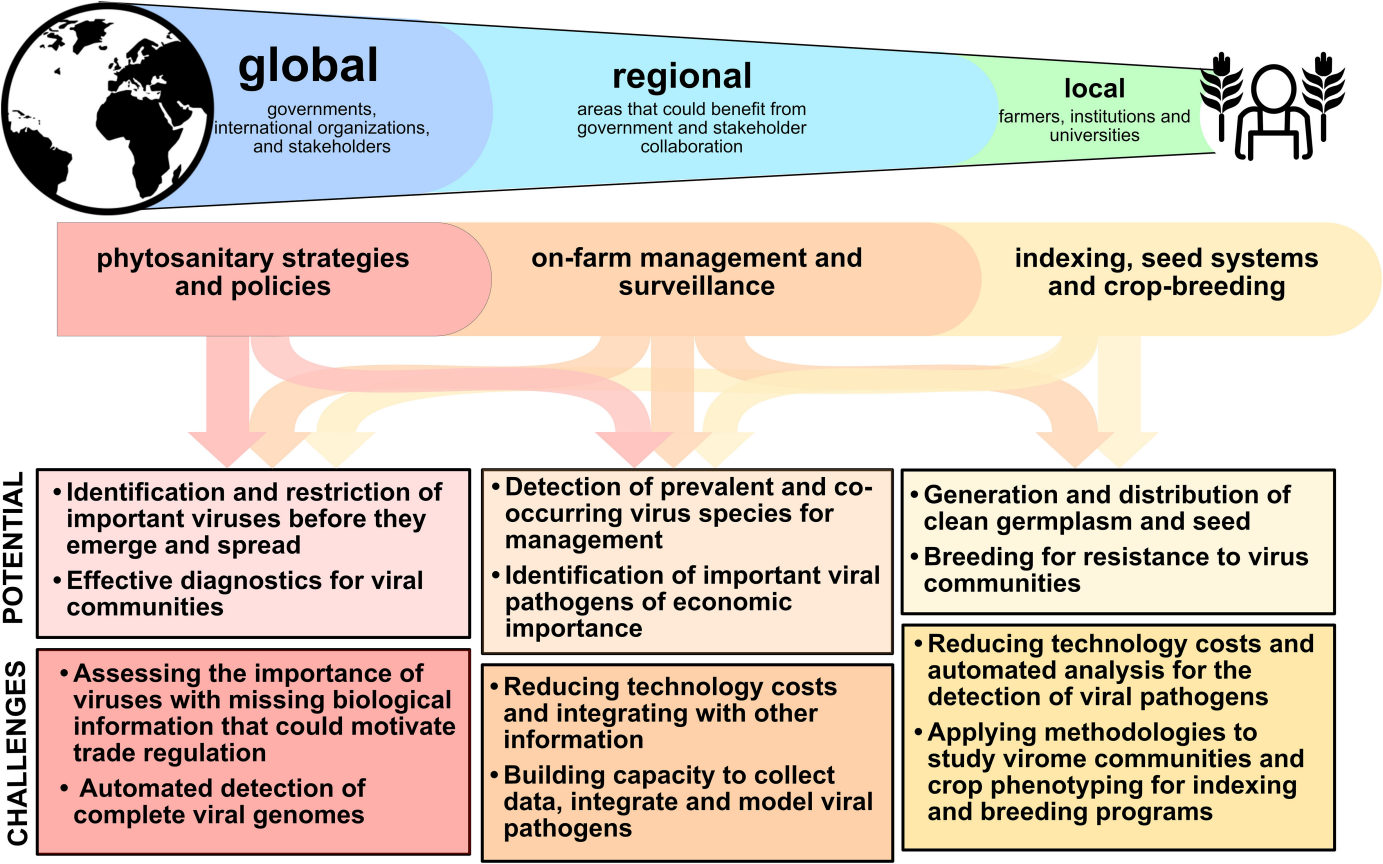
Virome analysis reveals complex interactions in mixed and single infections, with impacts from global to local scales. Translating information at each scale has both important potential as well as challenges for implementation. Effective translation can improve on-farm management, crop breeding and seed systems, and phytosanitary activities.

**Figure 2:**
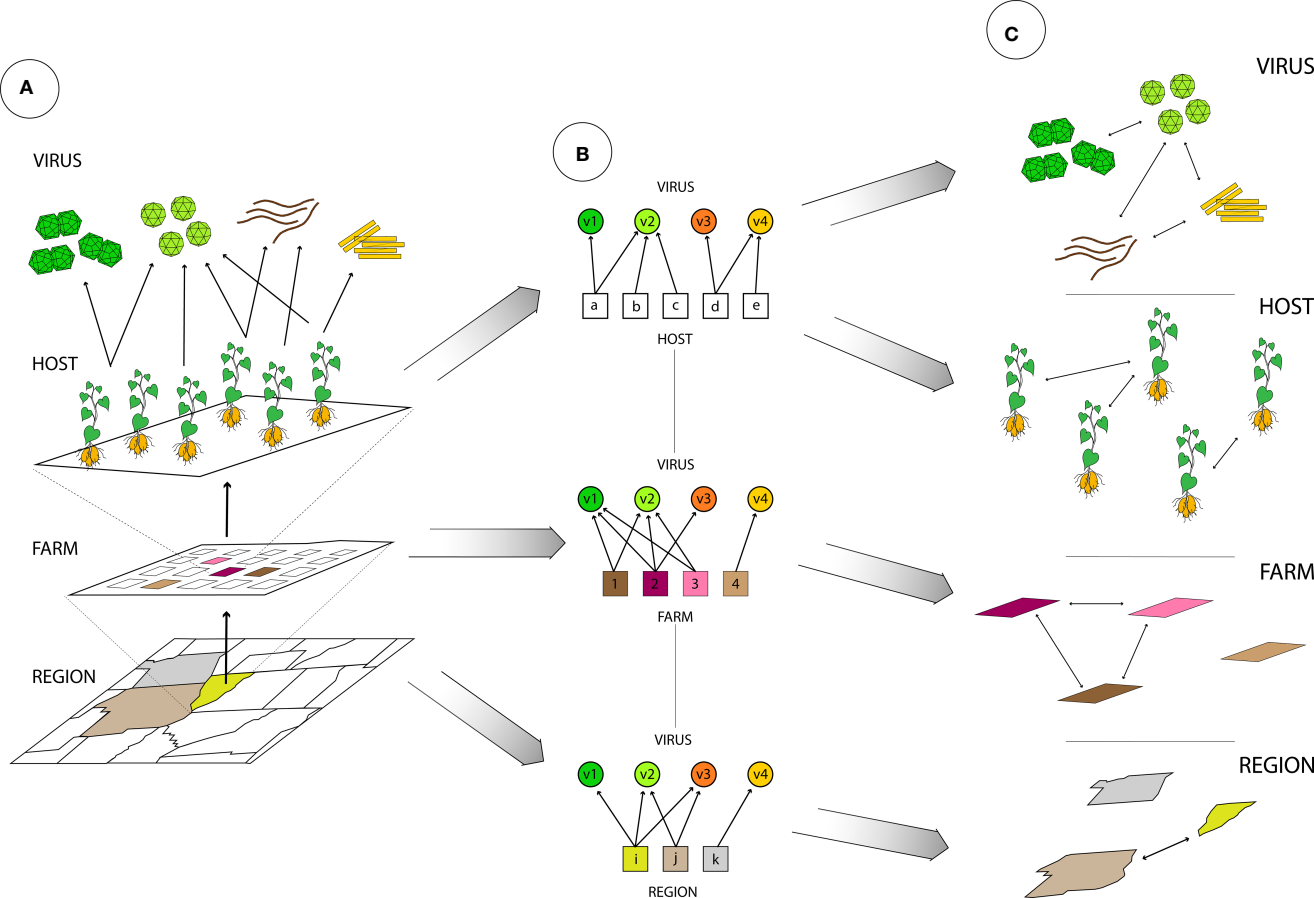
The spatial resolution of virome analysis, from low-resolution (counties and farms) to high-resolution (hosts), and virus associations at each scale. **(A)** Geographic representation of counties in a region, farms within a county, and hosts within a farm. Each layer could contain a different subset of viruses (a different virome). **(B)** Bipartite networks representing associations between two types of nodes, where circles represent viruses (v1 to v4), and where evaluation can be at multiple scales, for example: by region, where squares represent counties (i, j, k), by farm, where squares represent plots (1 to 4), and by host, where squares represent individual plants (a to e). **(C)** A one-mode network, where links between viruses represent their shared geographic or host associations and links between locations indicate similarity of their viromes, by region or by farm. Links between hosts represent similar viromes.

Our ability to characterize the virome of an individual plant, a plant community, or an ecological niche is a major scientific advance, creating many possibilities for translating virome information to improve crop disease management across scales, methodologies, and disciplines (**Figure 1**). A fuller analysis of the virome includes characterizing associations and potential interactions among viruses, virus distributions across host species, types of virus environments and changes across time. Complex virome datasets have been analyzed as ecological networks, with associations and ecological patterns such as host-virus and vector-virus associations and virus-virus co-associations (McLeish et al., 2019; Alcalá-Briseño et al., 2020), spatial distribution within the landscape (Alcala-Briseno et al., 2021; McLeish et al., 2021), and global and local movement (Alcalá-Briseño et al., 2020; Alcala-Briseno et al., 2021; McLeish et al., 2021; Baker et al., 2022). Network analysis can represent the interactions in these communities as links in a network, potentially representing virus species incidence in environments and other attributes such as relative abundance, means of transmission, vectors, virus-virus interactions, etc. (Luis et al., 2015; Poudel et al., 2016; Garrett et al., 2018). Analyses need to address long-studied ecological properties of viromes– such as species richness and other measures of diversity – and go beyond these to include structures that can be explored in network analysis, such as virus co-infections, host-virus distribution and assemblage, virus–vector interactions, and the host preferences of vectors (**Figure 2**). There is also potential to build on these network analyses by incorporating new network characteristics, such as including host phenotypic responses as variables in networks (Poudel et al., 2016), and including three-way and higher interactions among virus species (Battiston et al., 2020). These new types of data will inform strategies for monitoring emerging viral pathogens, when the data are available and accessible, and specialized software can be developed for real-time analyses.

New-generation diagnostic tools based on HTS offer extensive qualitative and quantitative information to understand virome ecological properties. This data can be analyzed using machine learning to help design precise intervention strategies. HTS has higher sensitivity that can help rapidly identify virus species that pose a risk for disease emergence, providing better understanding of previously unimportant viruses (Alcalá-Briseño et al., 2017; Fox et al., 2019) that may pose a critical risk when they co-occur with other synergistic virus species (Fox et al., 2019; Elvira Gonzalez et al., 2021). Improved methods have contributed to pathogen detection in plant diagnostic clinics, seed production initiatives, and ports of entry (Miller et al., 2009; Kumar et al., 2021), allowing the implementation of higher phytosanitary standards by companies and regulatory institutions for known and emerging pathogens (EPPO, 2021). However, novel viruses usually lack sufficient biological and epidemiological data to evaluate potential severity, transmission, and host range, complicating regulatory decision-making. To compensate for missing information, machine learning approaches can use classification algorithms to identify groups of greater interest and predict management strategies that could be the most effective treatment for a disease caused by one virus or several viruses, as we illustrate below.

Virome data, in terms of both virus incidence and associations, can be translated to target on-farm management strategies for particularly important viruses and virus combinations in a region. Ultimately, as sequencing becomes less and less expensive, diagnostic agencies and farm managers may screen their crop by implementing new generation diagnostic tools that can be used in surveillance strategies for viruses in agriculture and other systems (Boonham et al., 2014; Li et al., 2020b; Cassedy et al., 2021). Virome information across scales – farms, clusters of farms, and regions – could be used to customize management decisions given virus incidence and other ecological variables. Information about more complex systems – with factors such as secondary vegetation, intercropping, vectors, and varying crop genotypes and phenotypes – can be translated to inform management strategies, as illustrated for Mexican papaya orchards (Alcalá-Briseño et al., 2020). Virome network analysis can also reveal associations among multiple viruses within hosts or within regions (Garrett et al., 2018; Alcalá-Briseño et al., 2020; Cao et al., 2021), associations that could increase yield losses in local or global systems (Clark et al., 2012; Redinbaugh and Stewart, 2018; Kreuze et al., 2020).

Diagnostics generating new types of information can benefit crop breeding and seed system programs, testing whether germplasm is free of viruses and profiling viromes in resistant or tolerant genotypes that may be useful for further breeding and seed multiplication. Considering virome information can be especially important for breeding of vegetatively propagated crops, where the viral load accumulates in the host over time, reducing productivity and seed quality (Gibson and Kreuze, 2015; Thomas-Sharma et al., 2016; Jacobsen et al., 2019). When germplasm is shared among breeding programs, screening can benefit from virome analysis to ensure both known and novel viruses are not inadvertently exchanged. For crops propagated *via* true seed, exchange of germplasm among breeding programs is also complicated by seed-transmissible viruses, and could benefit from greater sensitivity of diagnosis (Dombrovsky and Smith, 2017; Kumar et al., 2019). Systems are needed to ensure virus-free germplasm for breeding programs to develop improved quality, yield, or resistance (or at least tolerance) to viral diseases that impact crop production (Kumar et al., 2019). Ultimately, virome science needs a systems framework for translating virome analysis, integrating information about virus ecology, evolution, and epidemiology to mitigate virus impacts on plant systems. Here we synthesize approaches that would be important components of this ongoing project.

## Biosecurity: Phytosanitary strategies and surveillance

### Phytosanitary strategies

Rapid identification of the causal agents of viral epidemics supports the development and implementation of phytosanitary measures for rapid containment or eradication. The timing of interventions is key to prevent the entry of exotic pathogens and slow down pathogen spread. For example, an outbreak of a new disease in yam bean was stopped in its tracks due to rapid identification (within a month) of the causal agent as a novel virus, yam bean mosaic virus, followed rapid amplification-based diagnostic development to eradicate infected plants and seed lots (Fuentes et al., 2012). Another example is early identification by HTS diagnostics of the causal agents that together cause maize lethal necrosis (MLN) in East Africa (Wangai et al., 2012; Mahuku et al., 2015), identifying maize chlorotic mottle virus (MCMV, *Machlomovirus*) and sugarcane mosaic virus (SCMV, *Potyvirus*) (Redinbaugh and Stewart, 2018). MCMV and SCMV can be seed transmitted, at low rates depending on the variety, and both are vectored by insects – Chrysomelid beetles in the case of MCMV and aphid species in the case of SCMV (Hilker et al., 2017; Redinbaugh and Stewart, 2018; Regassa et al., 2021). Diagnostics can indicate needed management strategies, however, smallholder farmers may not be able to afford inputs such as pesticides, certified clean seed and other management strategies (De Groote et al., 2020). A survey of farmers in Kenya from 2013 to 2018 reported a reduction in MLN by removing infected plants, implementation of resistant varieties and other control measurements (De Groote et al., 2020). The International Maize and Wheat Improvement Centre (CIMMYT) implemented rapid development of tolerant and resistant maize in Kenya, which likely helped to control MLN incidence (De Groote et al., 2020). However, despite efforts to train farmers and individuals on managing disease epidemics, there are still challenges to be addressed so that all farmers have access to needed information (Parsa et al., 2014; Buddenhagen et al., 2022; Streulens et al., 2022).

**Figure 3:**
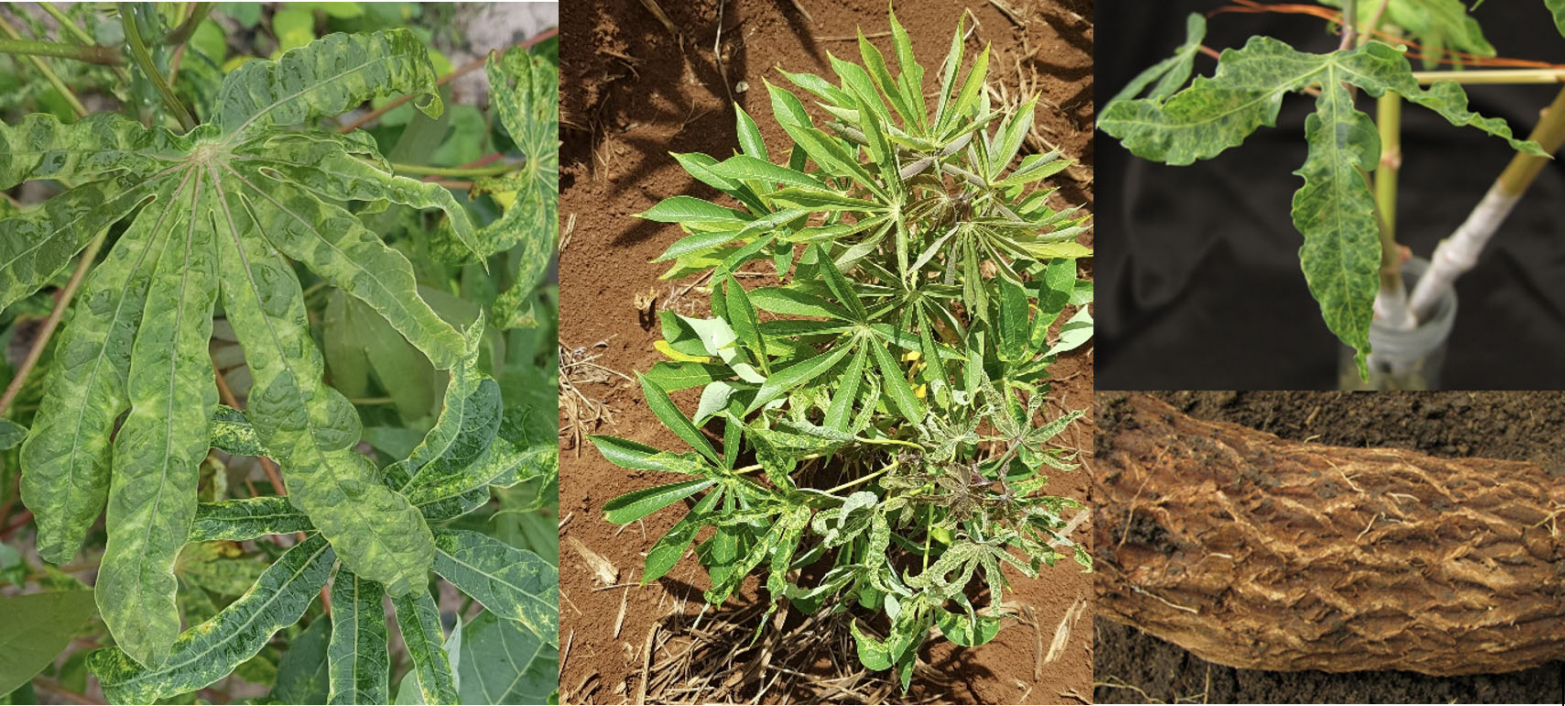
Left: Characteristic leaf symptoms of cassava mosaic disease (CMD), a disease associated with 12 different species of begomoviruses, affecting cassava in Africa, East Asia and Southeast Asia (Legg et al., 2015). Center: Cassava plant in Southeast Asia showing mixed symptoms of CMD (caused by Sri Lankan cassava mosaic virus) and cassava witches’ broom disease, a co-infection common in the region (Siriwan et al., 2020). Right: Leaf and root symptoms of cassava frogskin disease associated with a unique virome from the Americas (Pardo et al., 2022). Photos: W. Cuellar.

Another example where virome analysis can have an important role is the cross-continental epidemic of cassava mosaic disease (CMD, **Figure 3**). Cassava is a staple food crop in Sub-Saharan Africa and a primary industrial commodity in Southeast Asia. Cassava mosaic disease (CMD) causes yield losses reaching 30-50%, contributing to food insecurity and economic and social instability (Legg et al., 2011; Delaquis et al., 2018; Uke et al., 2021). In South Asia, single and mixed infections of two begomoviruses, Sri Lankan cassava mosaic virus (SLCMV) and Indian cassava mosaic virus (ICMV), cause CMD (Legg et al., 2011; Wang et al., 2016), while in Southeast Asia, CMD is currently caused by SLCMV alone. Specific assays would need specific primers to detect each virus and may miss co-infections, increasing the time and cost of processing, but HTS-diagnostics, in theory, can capture all viruses within a given sample. In the last five years, exchange of contaminated seed (stakes) and viruliferous whiteflies have spread SLCMV to Cambodia, Vietnam, South China, Thailand, and Lao PDR (Delaquis et al., 2018; Siriwan et al., 2020; Chittarath et al., 2021). Combinations of virus species may increase impacts on plants, and there may also be interactions between viruses and phytoplasmas or other bacterial and fungal pathogens. Surveillance of CMD in Southeast Asia and its whitefly vector (Leiva et al., 2022) is key to reducing spread of SLCMV and to timely detection if additional viruses are introduced, along with indexing programs to generate clean seed and use of resistant cultivars (Malik et al., 2022). Collaborations among national institutions and through international networks are central for the development and implementation of programs to establish clean seed production and management strategies to mitigate diseases (Kumar et al., 2019; Uke et al., 2021).

### Virome surveillance

Pathogen surveillance can be conducted with multiple interconnected goals, including detection of outbreaks, pathogen characterization, and geographic characterization – along with characterizing the current phytosanitary measures and quarantine regulations and whether they are sufficient (Carvajal Yepes et al., 2019; Kumar et al., 2019). Effective surveillance is based on data collection to determine the pathogen’s etiology and current distribution, and to parameterize epidemiological models of potential pathogen spread and management effects (Parnell et al., 2017; Garrett, 2021; Mastin et al., 2022). Eventually, virome surveillance will be based on routine collection of genomic and ecological data, building understanding and improving knowledge of emerging and re-emerging viruses in production areas (Carvajal Yepes et al., 2019). A range of methodologies can be used, including ELISA and PCR, however HTS-diagnostics also allows the detection of new or emerging viruses (Kumar et al., 2019), important for the identification of risk factors associated with viral emergence. Plant viruses are often asymptomatic and can spread unnoticed until significant economic losses have occurred (Uke et al., 2021). “Passive” virome surveillance based on samples submitted to plant diagnostic networks and disease clinics, and “active” virome surveillance based on sampling by phytosanitary agencies, researchers and other organizations, can both contribute to effective detection (Carvajal Yepes et al., 2019; Kumar et al., 2019; Iles et al., 2021; Kumar et al., 2021). Virome surveillance is crucial to attempts to eradicate and limit pathogens and to warn growers about new pathogens they may need to manage. Analysis of host distribution and disease dispersal across the agricultural landscape helps identify geographic priorities for surveillance and management interventions (Xing et al., 2020; Garrett, 2021). Researchers and international agencies continue to develop new diagnostic tools implementing machine learning and AI to provide early warnings of outbreaks and support prompt and effective responses (Pavan et al., 2011; Ouma et al., 2019).

Despite current phytosanitary standards and surveillance, emerging pathogens are disseminated *via* botanical seeds and vegetative planting material for distribution locally or globally, affecting smallholder farmers and large production areas. Recent proposals for a global surveillance system to mitigate pathogen introductions are being discussed (Carvajal Yepes et al., 2019). The increasing scalability and affordability of new sequencing technologies, combined with robotics and progress in nucleic acid extraction and enrichment, allow rapid virus identification (Ho and Tzanetakis, 2014; Roossinck et al., 2015; Massart et al., 2019). Integrating new diagnostic capabilities with well-matched sampling schemes will allow quick identification and mapping of abundance. Generation of large sequence data can be analyzed with classification algorithms supporting virus taxonomic classification. Species demarcation criteria are well established by the International Committee of Taxonomy of Viruses (ICTV), relying primarily on sequence similarity and the phylogenetics of genomes, genes or proteins (Simmonds et al., 2017). Additional analyses can be integrated using machine learning (Garrett et al., 2022), scaling up from the on-farm management examples discussed below. These advances support the development of a global surveillance system for plant disease, to mitigate the impact of emerging pathogens, to protect farmers’ livelihoods and food security.

## Risk assessment and on-farm management

### Virome risk assessments

Pest risk assessments (PRAs) evaluate the potential for introduction, establishment and spread of pathogens and pests, the magnitude of risk, and the economic impact for a specific region based on key risk variables such as environmental suitability, vector availability, crop area, and productivity (EFSA et al., 2018). The most common application of virome analysis has been in economically important crops such as maize, chili peppers, barley, grape, berries, and tropical fruits (Al Rwahnih et al., 2011; Jo et al., 2017; Jo et al., 2018; Wamaitha et al., 2018; Alcalá-Briseño et al., 2020; Saad et al., 2021). Potential disease severity and transmissibility are generally considered the most important traits when developing phytosanitary standards (Kumar et al., 2021), where pathogen epidemics are often more likely in areas with high monoculture density. Potential sources of viral dissemination are assessed, such as leaves, stems, flowers, fruits, and seeds that may move into a country. Seeds are the greatest concern since their distribution can rapidly disseminate an as-yet unidentified problem, such as the case of tomato brown rugose fruit virus (ToBRFV). ToBRFV originated in the Middle East and was disseminated globally within five years to nearly 50 countries, being eradicated in some parts of Europe, while many countries are working towards its eradication since it threatens tomato production worldwide (Salem et al., 2016; Luria et al., 2017; Oladokun et al., 2019; Batuman et al., 2020; Turina and Salem, 2022). In PRAs, the risk of contaminated plant material entering the territory is evaluated based on the current conditions in the region (EPPO, 2021). Using this type of information, National Plant Protection Organizations (NPPOs) and Regional Plant Protection Organizations (RPPOs) can determine whether the entrance of a virus or viruses should be regulated based on the potential impact of the virus when infecting alone and when associated with other virus species that already are present in the country.

PRAs need to account for global change factors such as climate and trade. Global climate change and extreme weather events drive crop losses, and can damage agricultural infrastructure, along with changing distributions of arthropod vectors and weeds that act as hosts (Garrett et al., 2020). When natural disasters destroy local infrastructure, there is particular risk that new pathogens will be introduced to the region; emergency needs make it difficult to implement effective sanitary measures (Lantagne et al., 2014). Informal seed exchange is a common practice globally that can disseminate infected plant material among neighbors and through entire regions (Delaquis et al., 2018; Andersen et al., 2019; Andersen Onofre et al., 2021; Nduwimana et al., 2022). The increase in international trade and e-commerce has also facilitated pathogen spread. ToBFRV, discussed above, is an example of these effects, being dispersed through certified international trade of contaminated seed (Oladokun et al., 2019; Van De Vossenberg et al., 2020). Ulluco tubers imported through e-commerce into the UK tested positive for viruses (Fox et al., 2019). Standardizing testing protocols across phytosanitary institutions, early warning systems, constant reassessment of PRAs, and promoting best agricultural practices such as using certified plant material, crop rotation, and resistant and tolerant plants would enhance phytosanitary measures.

The application of new diagnostic technologies raises a key question: which viruses among those observed in a plant virome should be regulated? The implementation of virome studies accelerated the discovery of viruses at a pace that surpasses the speed of experiments to characterize virus species and their host range. Phytosanitary regulations need to incorporate automated surveillance effectively in real-time, which represents a challenge in terms of methodologies, policies, and phytosanitary standards (Massart et al., 2017; Carvajal Yepes et al., 2019). The capacity of regulatory agencies may be overwhelmed by big data describing dozens or hundreds of species in each batch. Local agencies require bioinformatic capacity to develop efficient tools and expertise to interpret trends and highlight significant findings. Otherwise, there is a risk of paralyzing phytosanitary agencies, or conversely a risk of not making use of key information to address problem species before they spread widely. Automating diagnostics is necessary, and standalone practices for the analysis of genomic data to categorize by taxonomic identity have been implemented (Massart et al., 2019; Iles et al., 2021; Silva et al., 2021). Additional information – such as characterization of the relationships among viruses, modes of transmission, incidence, and co-infections – can be incorporated into machine learning for phytosanitary regulation.

### On-farm management strategies

Our understanding of the ecology of viruses across scales is growing, from individual samples to studies comprising multiple host, farms, and regions. This information can be complemented with agroecological metadata for a geographic location, cultivar, growth stage, weather, and other variables, generating large datasets. For example, individual plant samples can reveal virus-virus interactions that may explain symptomatology (Escalante et al., 2018; Okada et al., 2018). Surveillance of multiple host species and vectors could indicate which alternative hosts are important virus reservoirs and which weed species require active management to protect crops (Ng et al., 2011; Alcalá-Briseño et al., 2020; Mansouri et al., 2021). Monitoring viromes in farms and regionally could provide advance warning of virus incidence and potential disease outbreaks for farm managers and phytosanitary agencies. Understanding the phylogenetics and phylogeography of each virus locally, regionally, and globally will allow for a better understanding of how viruses were disseminated or introduced (Fuentes et al., 2019; Fuentes et al., 2021a; Fuentes et al., 2021b; Fuentes et al., 2022). Effective management of seed degeneration in vegetatively propagated crops can be achieved through the integration of seed health assessments and pest risk evaluations (Thomas-Sharma et al., 2017). Decision-making is enhanced by insight into the viruses in crops and weeds, including information on their transmissibility, host range, and vectors. This information can be used to guide phytosanitary practices and eradication programs on farms. At a regional scale, scenario analysis can evaluate how virome traits, management options, and growers’ decision-making processes combine to influence whether policies are successful for reducing the socioeconomic impacts of crop epidemics (Gilbertson et al., 2011; Garcia-Figuera et al., 2021; Etherton et al., 2023).

Larger and more complex viromes with multiple layers of information can be analyzed with machine learning algorithms such as decision trees, Bayesian and neural networks, and other support vector machines (Kotsiantis, 2007; Liakos et al., 2018; Chouhan et al., 2019; Topçuoğlu et al., 2020; Garrett et al., 2022). Virus characterization and taxonomic assignment are based on sequence similarity, serological properties, and other biological information such as transmission types, vector species, etc. (King et al., 2011; Lefkowitz et al., 2017; Simmonds et al., 2017). Machine learning approaches can help identify viromes that likely require similar management strategies, which we define here as “virome management units” (VMUs, see **Box 1**). VMUs are an analog to operational taxonomic units but based on information about management requirements rather than simply based on taxonomic similarity. Categorizing the potentially dozens of virus species in a virome into a smaller number of VMUs may make management more practical, reproducible, and potentially more automated and effective. Designing VMUs may be particularly useful in more complicated scenarios, such as managing viromes in intercropping systems, whole-farm virome management across multiple crop species and rotation schemes, or managing regional viromes across high cropland diversity.

**Figure 4:**
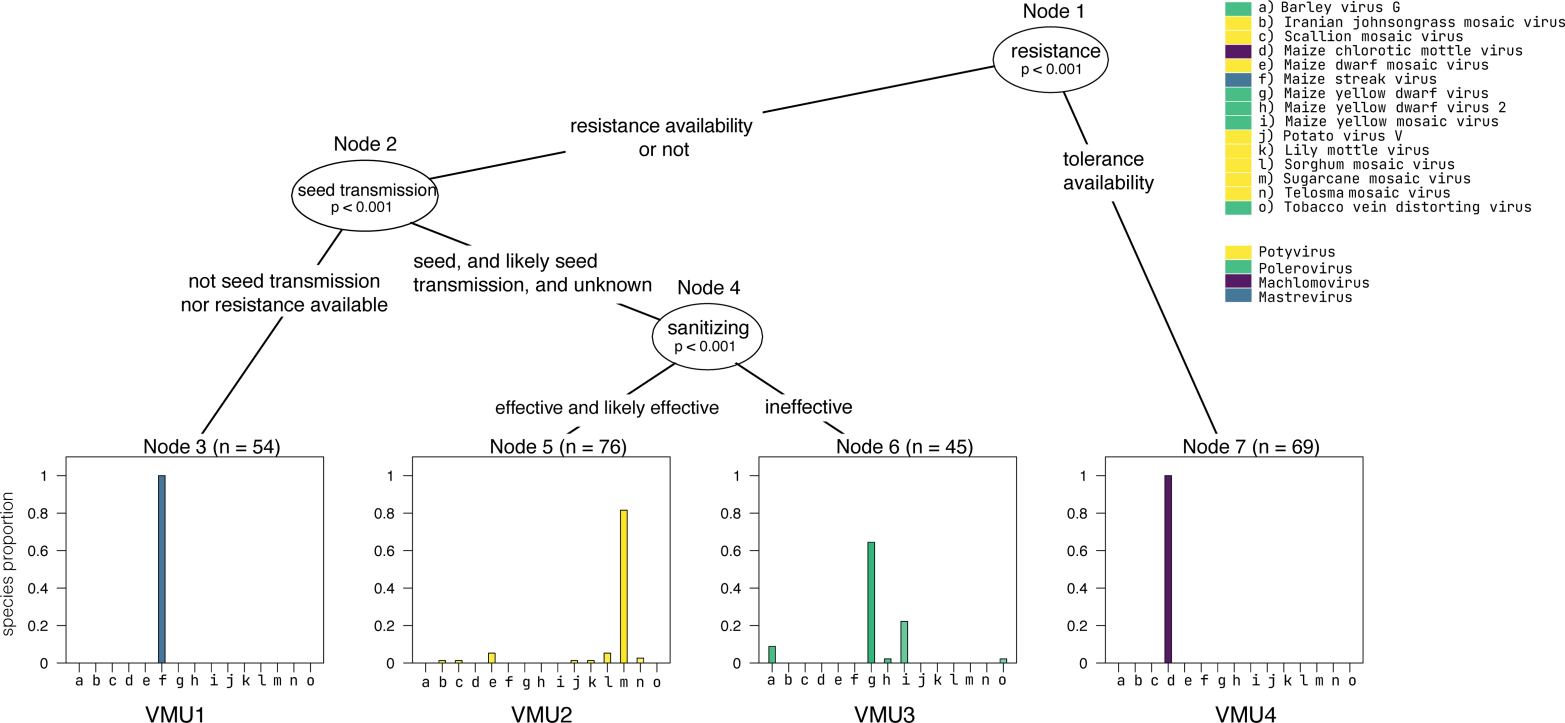
Virome management units associated with maize lethal necrosis, as described below.

As a simpler illustration of the potential for the use of VMUs (**Box 1**), we discuss an example for a single host species, based on the virome associated with MLN in Kenya reported by Wamaitha et al. (2018). Subsets of the virome are divided based on taxonomic information and management metadata, illustrating candidate management units. VMU characteristics may include degree of seed transmissibility, vector type, degree of mechanical transmission, and the effectiveness of potential management options: sanitation, use of clean seed, antifeedants and insecticides, and resistant (or at least tolerant) cultivars, if available. Decision trees can be used to divide virus species in virome data, starting with a root node, repeatedly partitioning the data into sub-groups represented as branches (**Figure 4**). Once a model has been trained, tested, and validated, it can be used to predict output classes for new virus species in terms of which VMU is the best fit for each species.

Box 1 **On-farm management based on grouping viruses in virome management units.**

Machine learning can identify groups of viruses that can be managed together according to their most effective control strategy. We use the term “virome management unit” (VMU) to refer to a subset of viruses within a virome that have similar characteristics relevant to management needs. This concept can be used to simplify management planning for viromes with many virus species, such as in the context of whole-farm or regional virome management. VMUs can be evaluated based on information about virus transmission, vector types, and management strategies used for each virus and their effectiveness, including tools such as sanitization, insecticides, and resistant or tolerant cultivars. As an illustration, consider the virome associated with MLN in Kenya (Wamaitha et al., 2018), with 15 virus species reported in four genera – *Potyvirus*, *Polerovirus*, *Machlomovirus*, and *Mastrevirus.* In this example, potyviruses and machlomoviruses are vectored by aphids and beetles, respectively, and both groups are mechanically transmitted. Poleroviruses are only aphid-transmitted but not mechanically transmitted. Seed transmission is reported for potyviruses and machlomoviruses, but not for poleroviruses and mastreviruses. Sanitation would work for mechanically transmitted viruses like potyviruses and machlomoviruses, but not for poleroviruses, while using virus-free seed would contribute to management of seed-transmitted viruses. Lack of information represents a challenge during classification, such that some VMUs may be defined in the short-run without classification information.

In this illustration of the VMU concept, VMUs were delimited using a supervised machine learning algorithm in R (R Core Team, 2022), a decision tree algorithm in the *party* package (Hothorn et al., 2006). In this decision tree, ovals represent a variable relevant to management that was used to categorize viruses into VMUs. Each rectangular node represents the decision output category, the VMU to which a virus or group of viruses is assigned. The MLN virome from Kenya is based on 244 maize samples, and *n* is the total number of entries that were assigned to each decision node (e.g., *n* = 54 in Node 3). Four distinct VMUs were identified in this maize virome. VMU1 consists of maize streak virus, an African native grass virus. VMU2 includes only potyviruses. VMU3 includes the poleroviruses. VMU4 includes the machlomovirus MCMV. VMU2 which are seed transmitted and mechanically transmitted can benefit from use of certified virus-free seed and sanitation practices. VMU4 could primarily benefit from using varieties resistant or tolerant to the MCMV virus, and virus-free seed. In some cases, such as VMU1, more biological information is needed to identify effective management strategies with high confidence. Resistance genes were identified as relevant for MCMV in VMU 4; maize with tolerance to MCMV can help mitigate the effects of MLN epidemics. Although there are maize hybrids resistant to SCMV, based on resistance genes like Scmv 1 and 2 (Jones et al., 2018; Redinbaugh et al., 2018), this classification did not include MCMV and SCMV in the same management group. Practical applications of VMU classification will be strengthened by more extensive data and exploration of the best algorithms for classification.

## Virome indexing, seed systems, and crop breeding

### Virome indexing for germplasm and seed systems

**Figure 5:**
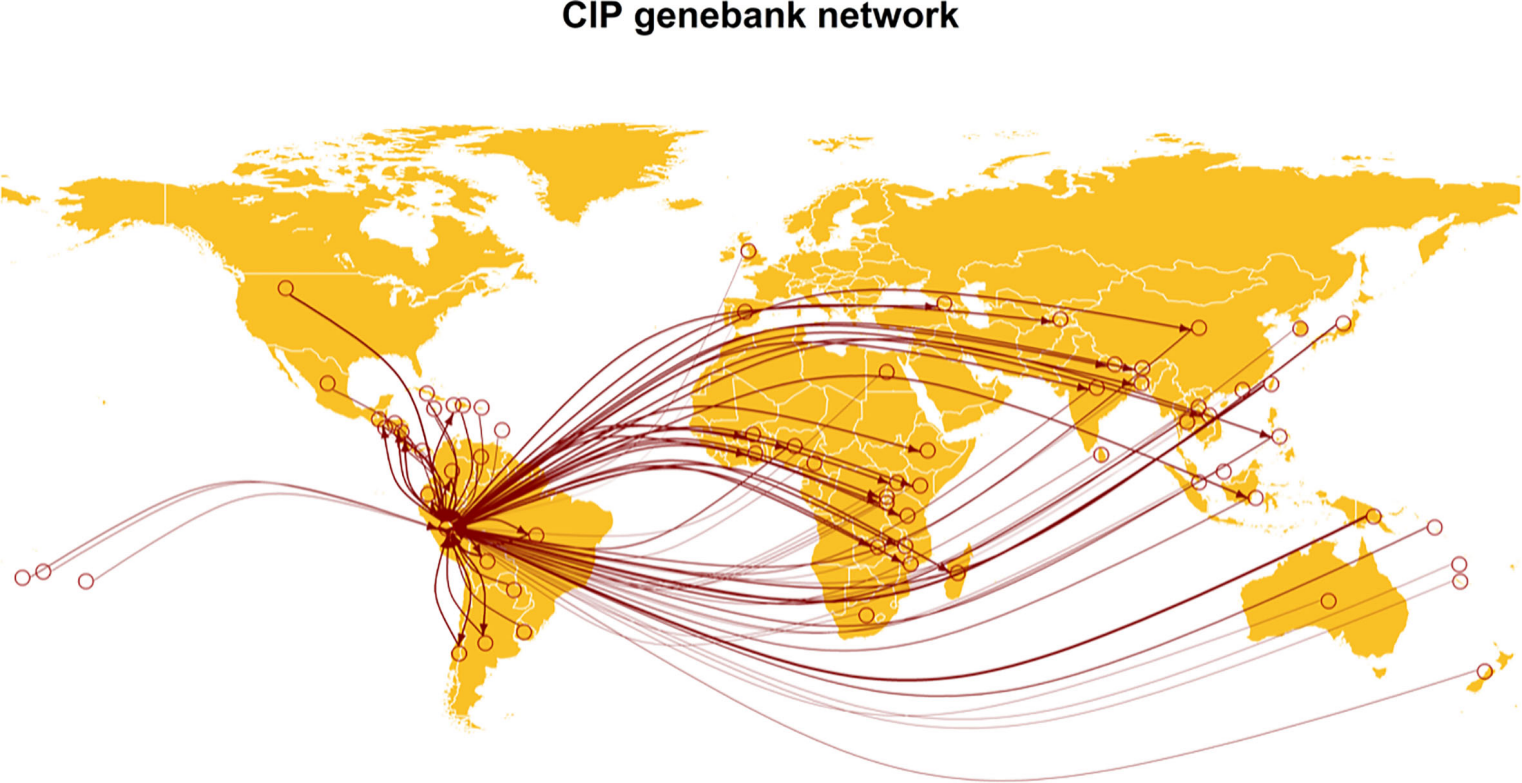
Thirty-two countries requested germplasm material for potato, sweetpotato, and Andean roots and tubers in 2020 from the Genebank of the International Potato Center (CIP) in Lima, Peru (data retrieved from https://cipotato.org/genebankcip/process/distribution_acquisition/). Virome analysis can help to ensure that viruses that may endanger crop production are not present in germplasm.

Virus indexing is the testing of germplasm to ensure it is free of viruses, a particularly important step for vegetatively propagated crops and tissue culture propagation, especially when germplasm is moving between breeding programs and companies, and between countries. The most common virus indexing methods include grafting of plants on indicator hosts, use of serological methods such as ELISA, and molecular detection through conventional PCR and qPCR (Boonham et al., 2014; Kumar et al., 2019; Silva et al., 2021). One of the caveats of these detection assays is that they are usually designed to target specific known viruses, meaning that a limited number of pathogens is tested in certified clean seed programs. Traditional diagnostics assays often fail at detecting novel viruses and genetic variants; however, with the implementation of HTS, all viruses can be recovered, as discussed above (Roossinck et al., 2015; Simmonds et al., 2017). Both DNA and RNA viruses can be detected using virions, small RNAs, dsRNAs, or total RNA, and these methods can be implemented in bulk or individual samples (Kreuze et al., 2009; Alcalá-Briseño et al., 2020; Moubset et al., 2022). Generating virus-free germplasm through indexing programs to (re)establish clean seed for individuals, associations, and germplasm banks prior to movement is very important to avoid the spread of plant pathogens. For example, the International Potato Center (CIP), a CGIAR center, distributes hundreds of indexed germplasm accessions free of pathogens to more than 100 countries globally (**Figure 5**). National programs such as the US National Clean Plant Network (NCPN) work to provide high-quality and virus-free plant material by working in partnership with growers, researchers, and the industry, developing and implementing indexing protocols to prevent the spread of pathogens. The NCPN also provides diagnostic services and educational resources to help growers and industry professionals identify and manage plant viruses. Similar programs are in place in many countries globally.

When considering how to manage viromes in germplasm or seed systems, it is important to distinguish between virus species that are already present in a region and those that may represent new introductions. For those viruses that are already endemic in a region, quality declared seed that maintains viruses at a low level may be the best economic value when seed completely free of a virus is impractical to produce for the time being (Choudhury et al., 2017; Mastenbroek et al., 2021). It may also not be practical to purchase off-farm seed to replace all seed every year (Thomas-Sharma et al., 2017; Navarrete et al., 2022). Defining quality standards for quality declared seed represents a balance between making seed available at a reasonable price and reducing epidemic rates. Considering this balance for a virome, rather than for a single virus species, represents an interesting challenge. For species in a virome that are new to a region and offer a substantial threat, reducing seed levels to zero is key, along with tracking the potential spread of new species through seed systems, evaluating management strategies, and understanding how farmers may adapt to the new disease scenario (McQuaid et al., 2016; Almekinders et al., 2019; Andersen et al., 2019; Ferris et al., 2020; Andersen Onofre et al., 2021; Nduwimana et al., 2022). Movement of at least a limited amount of contaminated plant materials might be inevitable in some cases, although indexing programs and implementation of new diagnostic tools based on HTS could help limit new introduction of viruses and other plant pathogens.

### Breeding programs and viromes

Systems ensuring virus-free germplasm support trade and seed distribution and are particularly important for developing resistance to viral diseases and reducing their impact on crop yields. Germplasm sharing among breeding programs in regions where different viruses are present may have helped to spread viruses globally (Kumar et al., 2019), and new generation diagnostics complemented with virome analysis have been used to ensure known or novel viruses are not exchanged (Massart et al., 2017). For crops that are propagated *via* true seeds, exchanging germplasm among breeding programs is similarly complicated by seed transmissible viruses (Dombrovsky and Smith, 2017). Screening germplasm to identify viruses is often especially important for breeding of vegetatively propagated crops where the viral load accumulates in the host over time, reducing the productivity or seed quality of crops (Gibson and Kreuze, 2015; Thomas-Sharma et al., 2016; Jacobsen et al., 2019; Andrade-Piedra et al., 2022). Indexing germplasm using HTS can also be used to generate gene expression profiles for testing how genotypes express resistance or tolerance, which can benefit breeding programs by providing recommendations for further breeding and propagation of clean seed. The development and use of high-throughput disease phenotyping facilitates such new approaches to understanding the impacts of viruses in different host genotypes. Ultimately, crop breeding may address resistance to viromes rather than to only one or two viruses, when the viromes of agroecological zones have been characterized and methods are available for exposing breeding materials to the relevant viromes.

HTS in crop breeding has been used to fingerprint the genomes of plant accessions and to characterize the diversity of breeding populations (Bhat et al., 2016; Vlk and Řepková, 2017). For example, HTS has been used to identify markers for resistance to viruses, where the genetic markers are used in genomic selection and genome-wide association mapping, and have been associated with differential expression in RNAseq experiments (Bhat et al., 2016; Vlk and Řepková, 2017; Bhat et al., 2021). Identification of markers by HTS in crops has supported development of resistance to pathogens such as cucumber mosaic virus (CMV), barley mosaic viruses, and potato virus Y (PVY) (Manivannan et al., 2018; Li et al., 2020a; Saidi and Hajibarat, 2021). HTS technologies that support virome indexing and fingerprinting of plant breeding populations, revealing important qualitative and quantitative traits, can improve plant breeding programs and seed systems. Gene expression responses to viral infections can be associated with resistance genes, such as the eIF4E recessive resistance gene in barley (Shi et al., 2019). PAL1 is another example of an overexpressed gene that confers resistance to cassava brown streak disease (Kavil et al., 2021). Complex interactions can sometimes protect crops, such as the tripartite interaction in papaya, which generated tolerance to papaya ringspot disease through an antagonistic effect of PapMV that elevated the expression of a pathogenesis-related protein (PR-1), reducing PRSV RNA accumulation (Chávez-Calvillo et al., 2016). Single or bulked samples have also been successfully used to identify resistance genes, and viruses (Roossinck et al., 2015; Shi et al., 2019; Kavil et al., 2021). Phenotypic and genotypic data can provide information for the development of disease-resistant cultivars, but the genetic structure underlying disease resistance regulatory networks is generally not simple. Identifying effective markers relies on the integration of good data for both phenotypes and genotypes to identify genomic regions for breeding (Bhat et al., 2016; Dasgupta et al., 2021).

## Discussion: Realizing the full potential of virome analyses

Undoubtedly the role of HTS-diagnostics and virome analysis in plant pathology will continue to expand in the future. But to have an impact on plant viral epidemics in real world settings, more is needed than technology alone. Frameworks for technology application must consider supporting factors including local access to equipment, computing power and data storage capacity, and staff capacity building for the interpretation of the complicated outputs of modern bioinformatic tools.

Australia and New Zealand, island nations with unique endemic biota, apply stringent biosecurity practices to reduce catastrophic introductions of invasive species (Simberloff, 2019; Pysek et al., 2020). New Zealand has an effective seed screening protocol with only an estimated 1.9% of lots being contaminated, but containing over 190 genera of weeds (Rubenstein et al., 2021), where weed seed also has the potential to introduce new viruses detrimental to crop production or natural plant systems. Virus screening in Australia employs a combination of visual methods, bioassays, ELISA, PCR and qPCR approaches, in which thresholds for acceptable viral contamination are determined through a regularly adjusted ‘appropriate level of protection’ evaluation (Whattam et al., 2021). As elsewhere, HTS approaches are undergoing evaluation. Although HTS approaches have long been touted as a powerful solution to post-entry screening (Rodoni, 2009), authorities must grapple with the challenges, including developing thresholds and quarantine lists for viral contamination, and protocols for dealing with novel virus pathogens. In the United States, HTS viral indexing has been used in a ‘provisional release propagation’ practice to allow growers to begin bulking imported materials in delineated areas while traditional indexing methods confirm the results (Villamor et al., 2019).

Another global trend is the emphasis on reforestation to sequester carbon and provide other ecosystem services (Brancalion et al., 2020; Holl and Brancalion, 2020). Related programs such as Sembrando Vida in Mexico and Central America are designed to mobilize planting material for cropping systems and forestry (Secretaria Del Bienestar, 2020). The field of restoration ecology also is based on movement of planting materials, to restore what had been the previous plant community (Harrington, 1999; Erickson and Halford, 2020). For example, the National Seed Strategy of the US Bureau of Land Management addresses use of native seed (National Academy of Sciences Engineering and Medicine, 2023). All these projects include potentially large-scale movement of plant materials that are sometimes subject to little analysis of associated pathogens. In the past, detailed consideration of pathogens was impractical, and it may still be impractical in many cases, such as restoration projects that include many wild plant species whose pathogens have not been studied. As virome analysis becomes a more practical tool, there will need to be important decisions about which viruses can be present in these projects. Some viruses may have important roles in maintaining the composition of wild plant communities, while other viruses may disrupt communities and give an advantage to invasive plant species (Rúa et al., 2011; Roossinck, 2019). Other viruses that will be discovered may have little effect, and too much attention to their management may reduce the benefits of these programs. An effective balance will need to be found, using new virome information to guard against destructive spread of viruses while minimally disrupting restoration.

Access to tools and support for virome-based analyses is scarcer in low-income countries. Many countries still struggle with maintenance of conventional plant health surveillance and screening systems, which demand significant resources to operate, even for valuable commercial crops like cassava in Southeast Asia, a cash crop for more than 8 million farmers (Malik et al., 2020). International donor support was needed to convene stakeholders to stimulate regional cooperation, and the first publications reporting the spread of SLCMV in the region involved researchers with sequencing capacity from China (Wang et al., 2016), CGIAR Research Centers (Minato et al., 2019; Leiva et al., 2020; Chittarath et al., 2021), and Japan (Uke et al., 2018). Across the global South, HTS technologies currently are not widely adopted and are often still inaccessible, and there are serious barriers to the use of complex viral community data in phytopathology screening, highlighting the need for accessible analysis in the short run, and capacity building in the long run.

For now, a dose of realism and humility is required given the track record of adoption of current, comparatively simple methods, such as PCR-based checks, in countries with limited resources. Plant virus epidemics are increasingly a globalized phenomenon (Miller et al., 2009). Widespread adoption of virome-based methods opens a whole new level of possibilities for plant virus research, but for their effective implementation to address plant epidemics to be realized, global approaches are needed. Open access software pipelines (e.g., Nextflow, Python, R and Linux) and repositories of viral sequence data (e.g., NextStrain) should be promoted. Machine learning and other AI approaches have unprecedented power to process this huge volume of data into actionable outputs. But they can only supplement, not replace, the capacity of phytosanitary authorities, field technicians, and research staff who are the source of data inputs. Around the world these agents continually compete for government funds in a crowded marketplace of urgent needs. Virome analytics should also serve a primary function of raising awareness among policymakers and funding mechanisms of the realities of viral ecosystems and the implications for a globalized world should viromes go unmonitored – the recent public awareness of viral disease spread due to the COVID-19 pandemic may make policymakers more receptive to these issues.

Fully embracing virome technologies means not only the development of increasingly user-friendly tools for accessibility, but also dedication to long-term training and capacity building for implementers around the world, and supporting critical infrastructure for sampling, analysis, and interpretation. People remain at the core of the application of these technologies.

## Author contributions

RIAB and KG conceptualized the synthesis. All authors contributed content for the synthesis. RIAB and KG developed a final document with comments from all authors. All authors contributed to the article and approved the submitted version.
